# Genetics of Cholesterol-Related Genes in Metabolic Syndrome: A Review of Current Evidence

**DOI:** 10.3390/biomedicines10123239

**Published:** 2022-12-13

**Authors:** Sok Kuan Wong, Fitri Fareez Ramli, Adli Ali, Nurul ‘Izzah Ibrahim

**Affiliations:** 1 Department of Pharmacology, Faculty of Medicine, Universiti Kebangsaan Malaysia, Jalan Yaacob Latif, Bandar Tun Razak, Cheras 56000, Kuala Lumpur, Malaysia; 2 Clinical Psychopharmacology Research Unit, Department of Psychiatry, University of Oxford, Warneford Hospital, Oxford OX3 7JX, UK; 3 Department of Pediatrics, Faculty of Medicine, Universiti Kebangsaan Malaysia, Jalan Yaacob Latif, Bandar Tun Razak, Cheras 56000, Kuala Lumpur, Malaysia

**Keywords:** cholesterol-related genes, hyperlipidemia, insulin resistance, hypertension, obesity

## Abstract

Metabolic syndrome (MetS) refers to a cluster of metabolic dysregulations, which include insulin resistance, obesity, atherogenic dyslipidemia and hypertension. The complex pathogenesis of MetS encompasses the interplay between environmental and genetic factors. Environmental factors such as excessive nutrients and sedentary lifestyle are modifiable and could be improved by lifestyle modification. However, genetic susceptibility to MetS, a non-modifiable factor, has attracted the attention of researchers, which could act as the basis for future diagnosis, prognosis, and therapy for MetS. Several cholesterol-related genes associated with each characteristic of MetS have been identified, such as apolipoprotein, lipoprotein lipase (LPL), cholesteryl ester transfer protein (CETP) and adiponectin. This review aims to summarize the genetic information of cholesterol-related genes in MetS, which may potentially serve as biomarkers for early prevention and management of MetS.

## 1. Introduction

Metabolic syndrome (MetS) is characterized by a combination of at least three metabolic abnormalities, which include increased abdominal circumference, fasting blood glucose, blood pressure, triglycerides (TG) and reduced high-density lipoprotein (HDL) cholesterol [1]. These MetS features are important risk factors for premature cardiovascular disease. The pathogenesis of MetS is rather complex and not fully understood, influenced by the interplay between environmental and genetic factors. In addition to excessive nutrients and sedentary lifestyle, which serve as the modifiable environmental factors, the genetic susceptibility on MetS has attracted the attention of researchers aiming to provide a better understanding on the disorder.

Dyslipidemia is the major constituent of MetS, characterized by raised free fatty acids (FFAs), TG, small dense low-density lipoprotein cholesterol (LDL-C) and apolipoprotein B (apoB) levels, but low HDL cholesterol level [2]. Insulin resistance, which is associated with increased fasting blood glucose, increases the visceral adipocyte’s sensitivity toward lipolytic hormones [2]. These conditions result in a flux of FFA to the liver, further stimulating hepatic TG synthesis and subsequently promotes ApoB formation [2,3]. Meanwhile, for the formation of LDL, an important parameter in dyslipidemia is mediated by lipoprotein lipase (LPL) in muscles and adipose tissues [2]. These processes might indicate the interaction of various cell structures in the development of MetS and could influence each of the MetS features in multiple ways. There are various candidate genes essential in regulating lipid metabolism have been identified. For instance, adiponectin gene variants with T alleles at rs1501299 were correlated with lower HDL cholesterol in hypertensive subjects [4]. The overexpression of peroxisome proliferator-activated receptor-gamma (PPARγ) decreased cholesterol levels via multiple pathways of lipid biosynthesis and metabolism [5]. Leptin stimulated the expression of lipoprotein lipase (LPL), promoted hepatic HDL cholesterol clearance, and its level was inversely corelated with HDL cholesterol and apolipoprotein A [6]. Cholesteryl ester transfer protein (CETP) is a key regulator for HDL remodeling, facilitating lipid transfer among lipoprotein classes and reducing HDL cholesterol levels [7].

In this review, the role of cholesterol-related genes in the development and progression of MetS will be reviewed accordingly to its characteristics of dyslipidemia, obesity, hyperglycaema and hypertension. We hope to provide the readers with an overview on the genetic information of cholesterol in MetS, which may potentially serve as biomarkers for early prevention and management of MetS.

## 2. The Role of Cholesterol-Related Genes in Dyslipidemia

### 2.1. Apolipoprotein

Lipids, including cholesterols and TG, play vital roles in many physiological processes [8]. The cholesterols form a part of the plasma membrane and regulate membrane properties, such as thickness, internal curvature and permeability [9]. Cholesterols are also required to synthesize various molecules, such as bile acids and steroid hormones, and function as a regulator in neuronal signaling pathways. On the other hand, TG is the energy source for muscle and adipose tissues. Given the hydrophobic characteristics of TG and cholesterols, the molecules are transported in lipoproteins and chylomicrons. The carriers consist of a core hydrophobic containing a variable amount of cholesterol esters and TG enveloped by phospholipids, free cholesterol and apolipoproteins [8]. Although essential, abnormal lipid levels, commonly described as dyslipidemia, are detrimental in that it increases the risk of many diseases, including cerebrovascular and cardiovascular diseases [2,8]. Dyslipidemia can be characterized by an abnormal value of TG of ≥1.70 mmol/L, HDL of <1.03 mmol/L for males and <1.29 mmol/L for females, respectively [10]. Although the syndrome increases the risk of cardiovascular diseases, each component of the lipid profile is an independent risk predictor for MetS. Numerous population studies reported that TC, LDL and HDL are independently correlated with various cardiovascular diseases, including myocardial infarction [11,12].

Apolipoproteins play a pivotal role in TG and cholesterol transport and metabolism. Apo B is a major protein component of all types of pro-atherogenic lipoproteins, including very low-density lipoprotein (VLDL), intermediate-density lipoprotein (IDL) and LDL [13]. There are two Apo B types, Apo B100 and Apo B48, encoded by the same gene. However, their molecular sizes are different. Apo B48 is smaller and has a relative molecular weight of approximately 48% of the Apo B100, as determined by sodium dodecyl sulphate gel electrophoresis [14]. Moreover, Apo B48 is synthesized in the intestines, constituting the primary apolipoprotein present in chylomicrons [14,15]. In contrast, Apo B100 is predominantly expressed in the liver, although a small fraction of Apo B100 is expressed in the intestines [15].

The gene encoding Apo B, known as the *APOB* gene, is located in the short arm of chromosome 2 [16]. The gene is 43 kb long and comprises 28 introns and 29 exons variably scattered in three thrombolytic peptides (T2, T3, T4) [17]. The intron interruptions on the coding sequences occur mainly in peptide T4, followed by T2 and T3, with the intron amounts 24, 3, and 1, respectively [17]. Variable lengths of introns and exons have been determined, ranging from 107–3000 and from 39–7572 base pairs, respectively [17]. To date, more than 18,500 SNPs records of the *APOB* gene are available in the National Library of Medicine [18].

The elevation of Apo B is an essential cardiovascular disease biomarker [19]. A recent mendelian randomization study reported that high Apo B predicted a lower life span and a more significant risk of heart disease [20]. Various single nucleotide polymorphisms (SNPs) in the *APOB* gene cause amino acid substitution or loss of restriction sites by affecting specific endonucleases, such as *Rsa*I and *Eco*RI. Other SNPs cause no changes in translating the same amino acids, a condition known as silent mutation [17]. Although there are no changes in amino acids, silent mutations of specific SNPs are attributable to increased cardiovascular risks [21]. For instance, an rs693 polymorphism has been reported to cause an altered lipid profile [16,21,22]. The SNP rs693 is located on the most extended exon of the *APOB* gene, with 7572 base pairs [17]. Two allele forms, C- and T-alleles, contribute to polymorphism in rs693. The latter is a minor allele, postulated as the risk allele [16]. The presence of the T-allele changes the genetic code. However, the same amino acid, threonine, forms during translation. Interestingly, the rs693 silent mutation is associated with various changes in metabolic profile [16].

A meta-analysis conducted by Niu et al. (2017), which included the articles published before December 2016, had reported a positive association between the rs693 T-allele carrier with TC (*n* = 41,764), TG (*n* = 22,128), LDL (*n* = 22,286) and Apo B (*n* = 12,364) levels than the non-T-allele carrier. In contrast, the T-allele carriers of rs693 negatively correlated with HDL (*n* = 39,292) levels compared to non-T-allele carriers [16]. Numerous studies published from 2017 onwards reported inconsistent results, secondary to various limitations, including sample size, age and comorbidities. Although multiple limitations exist, the discussion of individual studies provides different perspectives regarding specific populations. Table 1 summarizes the genetic studies that address the association between specific polymorphisms and lipid parameters in the specific populations.

A study in an elderly Brazilian cohort reported significant associations between SNP rs693 and TC, total lipid and LDL levels, with homozygous TT demonstrating higher levels than the C-allele carriers [21]. Moreover, a case-control study on an Iranian population reported that T-allele carriers had a significantly higher risk of familial hypercholesterolemia than the non-carriers [23]. Familial hypercholesterolemia is a common genetic disease, affecting 1 person in 311 globally [24]. Cardiovascular disease is estimated to develop in 1 in 17 individuals with familial hypercholesterolemia [24]. Apo B polymorphism is one of three primary defects attributable to familial hypercholesterolemia [25]. Genetic testing in the disease is essential for risk prediction. It facilitates the prevention and treatment plans for individuals at risk of cardiovascular disease.

Alghamdi et al. (2021) reported a significant association between the rs693 AG genotype and TC levels and the rs693 GG genotype and TG levels in a young female group with MetS compared to controls; however, there was no significant association in other genotypes (AA/AG/GG) and lipid parameters, including LDL, HDL and Apo B100 levels, between the MetS and control groups [22]. Similarly, a case-control study involving patients with acute coronary syndrome and healthy controls found no correlation between SNP rs693 polymorphisms and apolipoprotein B levels [26]. In contrast, negligible associations were reported in a study in a Colombian Caribbean population between T-allele and C-allele carriers in TC, TG, LDL and HDL levels regardless of genetic models (dominant, recessive, co-dominant and additive) [27].

How silent mutation of rs693 affects lipid profiles remains obscured. A possible explanation is the association of the SNP rs693 with other alleles in the *APOB* gene or other genes at different loci, a condition known as linkage disequilibrium [21,28,29]. For instance, Li et al. (2020) reported negligible associations between the SNP rs693 and multiple APOB genetic variations in a case-control study involving participants with and without obstructive sleep apnea [29]. Interestingly, positive associations with TC, LDL and Apo B were reported when rs693 was included in a group of Apo B SNPs. MetS components, including high TG and low HDL levels, are essential risk factors for obstructive sleep apnea [30]. To summarize, the SNP rs693 polymorphisms are linked with various components of metabolic syndrome, including LDL and HDL. In addition, SNP rs693 polymorphisms are associated with other lipid parameters, including TC, TG, total lipid and Apo B100.

Another SNP in the *APOB* gene widely studied is rs17240441, located in exon 1, which consists of 210 base pairs [16,17]. Two allele forms, insertion (ins) and deletion (del), contribute to the polymorphism of rs17240441. The possession of the del-allele causes deletion of the nine-nucleotide sequence (GCAGCGCCA), resulting in the loss of 3 (leucine-alanine-leucine) out of 27 amino acid residues [16]. The removal may alter the hydrophobicity levels and Apo B processing as the sequence might be located in the leader peptide region [31]. The entry and translocation across the plasma membrane of the proteins are two stages regulated by the leader peptide before protein release. The structural changes due to genetic polymorphism may affect the export process, particularly in the translocation process, resulting in altered Apo B processing and export [32].

Cardiovascular risks are attributable to the del-allele [33]. A meta-analysis study involving 23 studies reported higher levels of TC (*n* = 7875), LDL (*n* = 5658) and Apo B (*n* = 5047) in the del-allele carriers than in the non-del allele carriers (I). However, negligible associations were reported in TG (*n* = 7411) and HDL (*n* = 5124) levels [16]. Moreover, associations between the SNP rs17240441 and lipid profile are reported in various populations, including a group of teenagers with essential hypertension (with or without hypercholesterolemia) [34], human-deficiency virus (HIV)-infected patients on anti-retroviral treatment [35] and healthy people [36].

Higher levels of TC in the del/del genotype than the ins-allele carriers were reported in the teenagers with essential hypertension both with and without hypercholesterolemia. Nevertheless, the genotype del/del was associated with a higher LDL than the ins-allele carriers only in the teenage group with essential hypertension without hypercholesterolemia [34]. In contrast, Vimaleswaran et al. (2015) reported that ins-allele carriers had significantly higher fasting TC, TG and LDL but lower HDL compared to homozygotes del/del. Interestingly, measurement of post-prandial TG in the same study found that homozygotes ins/ins had significantly higher TG than the del-allele carriers [36].

In HIV-infected patients on anti-retroviral treatment, higher levels of TC and LDL were reported in the rs17240441 genotype del/del than the genotype ins/ins adjusted for age, gender and lipid-lowering agents use. However, only LDL levels remained significant after multiple testing corrections [35]. Previous studies demonstrated that the protease inhibitors (e.g., indinavir and ritonavir) used in HIV-infected individuals altered lipid profiles as characterized by increased TC [37,38], TG [37,38] and LDL [37]. However, the genetic study reported no significant association between rs17240441 polymorphisms and lipid profiles [35]. One mechanism of protease inhibitors-induced lipid abnormalities is due to endoplasmic reticulum stress and autophagy inhibition in adipocytes, resulting in lipid metabolism abnormality. Deranged lipid profiles, characterized by low HDL levels but high TG levels, are components of metabolic syndrome. These findings supported the role of the SNP rs17240441 in the pathophysiology of metabolic syndrome via the modulation of various lipid components, including Apo B, TC and LDL.

Opposite to Apo B, which is the primary apolipoprotein found in pro-atherogenic lipoproteins, Apo A1 is the primary component of an anti-atherogenic lipoprotein, HDL [39]. The Apo A1 protection against atherogenesis is attributable to its inhibitory effects on platelet aggregation via the synergistic effect with prostacyclin [39,40]. Stabilization of the prostacyclin by Apo A1 enhances the anti-aggregatory effect, preventing thrombus formation at the injured vascular loci [39]. In addition, Apo A1 plays an essential role in the reverse cholesterol transport from peripheral tissues back to the liver via interactions with various receptors [39].

Other common SNPs contributing to dyslipidemia are located in the *APOA5-A4-C3-A1* gene complex or near the complex. The SNP rs964184 is located near the complex [41]. Woestijne et al. (2014) reported that G-allele (minor allele) carriers had significantly higher TG levels and Apo B but lower HDL levels. Furthermore, the study found BMI as a predictor for TG levels in heterozygous genotypes of rs964184 [41]. Moreover, an interregional study involving more than 100,000 people of European origin in the US, Europe and Australia reported that rs964184 was significantly associated with numerous lipid parameters, including TC, TG, LDL and HDL levels [42]. However, a meta-analysis of non-European cohorts reported mixed results. Only HDL and TG levels were significantly associated with SNP rs964184 in the East Asian cohort (*n* = 15,046), and TG levels were the only lipid parameter associated with SNP rs964184 in the South Asian cohort (*n* = 9705). In contrast, no significant association was found between SNP rs964184 and lipid parameters in the African American group (*n* = 8061) [42].

Replicative studies in other populations in China further supported the role of SNP rs964184 in lipid parameters [29,43]. A study in the obstructive sleep apnea population found a significant inverse association between SNP rs964184 and Apo A1 levels [29]. Interestingly, more significant correlations were reported when SNP rs964184 was analyzed as a cluster with other Apo A1 SNPs, with positive associations with HDL, LDL and Apo A1 levels but a negative association with TG levels [29]. Qiu et al. (2018) conducted a study on Han and Maonan Chinese populations, two populations with distinct characteristics in terms of geographical terrain, cultures and lifestyles. The study reported lower HDL levels in the Maonan Chinese group with the risk allele carrier. An inverse correlation was only reported in the male subgroup [43]. In contrast, a positive association between the risk allele carrier of rs964184 and TG levels but negative associations with Apo A1 levels and Apo A1/Apo B ratio were reported in the Han Chinese population. Intriguingly, the significance of associations changed during subgroup analyses, with TG levels remaining significant for females and the Apo A1 levels and Apo A1/Apo B ratio for males. However, association direction (positive or inverse) was not affected [43].

Due to the fact that we spend most of our awake time in a post-prandial state, Wojczynski et al. (2015) performed a genome-wide association study in European origin and Amish populations to investigate the role of genetic variants on post-prandial TG levels. In that study, they found a remarkable association between rs964184 and post-prandial TG levels. Interestingly, the significance association diminished when the baseline TG values were controlled, suggesting that SNP rs964184 is the primary determinant for baseline rather than post-prandial TG levels [44]. Similarly, Alcala-Diaz et al. (2022) reported higher post-prandial TG levels in the risk G-allele carrier at baseline. A three-year dietary intervention with a low-fat diet significantly lowered the post-prandial TG levels, comparable to the CC genotype [45]. The finding suggests that SNP rs964184 can be modulated by environmental factors, such as diet in the study, possibly through the gene–environment interaction.

The mechanism of how SNP rs964184 affects the lipid parameters might be attributable to the location of the SNP in a three-prime untranslated region (3-UTR) of the zinc finger 1 (ZPR1) gene [45]. Although the part is not translated, the 3-UTR plays a critical role in the structural and functional aspects of mRNA and proteins [46]. The cholesterol regulation of the ZPR1′s promoter part is attributable to the capability of the region to interact with peroxisome proliferator-activated receptor gamma (PPARG) proteins 1 and 2 [45]. PPARG1 is expressed in most tissues, but PPARG2 is primarily expressed in adipose tissue [47]. Without stimulus, the PPARs present as a complex with a co-repressor molecule. Activating PPARs with specific ligands promote the PPAR-coactivator complex to bind to the DNA promoter region, such as the ZPR1 region, resulting in the activation or inhibition of specific genes [45,47]. For instance, the genes activated may be essential in cholesterol metabolism through activating hepatocyte nuclear factor-4 alpha [45,48]. In summary, SNP rs964184 polymorphisms correlate with various lipid components, such as TC, LDL, Apo A1 and Apo B levels, as well as components of metabolic syndrome TG and HDL.

**Table 1:** The relationship between specific polymorphisms of apoliprotein and lipid parameters.

Study Type	Model/Population	Country	Genes Involved	Findings	Reference
Cross-sectional study	Females with and without MetS (*n* = 141; aged from 18–25 years).	Saudi Arabia	ApoB100 rs693	ApoB100 rs693 AG and GG genotypes were associated with higher TC and TG levels in the MetS group, respectively. NS: ApoB100 rs693 (AA/AG/GG) in HDL, LDL and TG between MS and control. ApoB100 rs693 (AA/GG) in TC between MS and control.	[22]
Cross-sectional study	Elderly outpatient participants (*n* = 644; aged ≥ 60 years).	Brazil	Apo B100 rs693	Significantly higher TC, total lipid and LDL-C in TT than C-allele carriers of rs693.	[21]
Case-control study	Patients with familial hypercholesterolemia (*n* = 120; aged 48.7 years) and healthy controls (*n* = 120; aged 41.4 years).	Iran	Apo B rs693 and rs515135	A significant positive association between T-allele carrier and familial hypercholesterolemia. HW between rs693 and rs515135.	[23]
Cross-sectional study	Colombian Caribbean healthy controls (*n* = 108; aged 52.0 years).	Colombia	Apo B Rs693	NS between T-allele and C-allele carries in TC, TG, LDL and HDL.	[27]
Case-control study	Russian teenagers with essential hypertension with or without hypercholesterolemia (*n* = 182; aged from 12–18 years).	Russia	Apo B rs17240441	A significantly higher TC in genotypes del/del than ins/del or ins/ins in teenagers with hypertension with and without hypercholesterolemia. A significantly higher LDL in genotypes del/del than ins/del or ins/ins in teenagers with hypertension without hypercholesterolemia.	[34]
Cross-sectional study	Healthy volunteers (*n* = 147; aged from 20–70 years).	United Kingdom	Apo B rs17240441	The ins/del and ins/ins genotypes had higher fasting TC, LDL and TG but lower HDL than del/del genotypes. The ins/ins had higher postprandial TG than ins/del and del/del genotypes.	[36]
Cross-sectional study	614 HIV-infected patients on antiretroviral treatment with undetectable viral loads (mean age of 43.0, 55.5% males, 57% Euro-descendants).	Brazil	Apo B rs693, rs17240441,	Significant associations between APOB rs693, rs17240441, TC and LDL.	[35]
Case-control study	300 patients with ACS and 300 healthy controls.	Mexico	Apo A1 and Apo B	Apo A1 SNPs rs670 and rs5070 and Apo A1 levels: NS. Apo B SNPs rs693 and Apo B levels: NS.	[26]
Prospective cohort	5547 patients with vascular diseases.	Netherlands	Apo A1 rs964184	Significant association between rs964184 and TG, HDL, non-HDL, Apo B.	[41]
Genome-wide association study	872 participants of European origin (mean age 50 years, 50.8% females) and 843 Amish participants (mean age 44 years, 46.3%).	United States of America	Apo A1 rs964184	Significant association between rs964184 and post-prandial TG levels. However, the significant levels diminished after controlling for baseline TG levels.	[44]
Cross-sectional study	4007 people with or without obstructive sleep apnea (aged from 29–53, 90.3% male).	China	Apo A1 and Apo B SNPs	Association between Apo SNPs and Apo levels Apo A1 SNPs and Apo A1 levels: (+) rs9804646; (−) rs964184. Apo B SNPs and Apo B levels: (+) rs1367117 (−) rs12713956, rs2854725 NS: rs693, Apo B: (+) rs2854725. Association between Apo GRS and biochemical parameters: ApoA1 (rs964184, rs9804646, rs10047462, and rs888246): (+) HDL, LDL, ApoA1; (−) TG. Apo B (rs1042031, rs693, rs2854725, rs1367117 and rs12713956): (+) TC, LDL, Apo B.	[29]
Cross-sectional study	867 Maonan (62.1% female) and 820 (62.7%) Han Chinese healthy volunteers (aged from 22–92).	China	Apo A1 rs964184	Maonan: Lower HDL in G allele carriers (both males and females). Correlation between genotype and HDL in males (−) Han: Higher TG in G allele carriers (total) and lower ApoA1 levels and ApoA1/ApoB ratio. Correlation between genotype and ApoA1 levels (−), ApoA1/Apo B ratio (−) in malesCorrelation between genotype and TG in females (+) Maonan and Han combined:Association between ApoA1 genotype and HDL (−) and ApoA1 levels (−).	[43]
Cross-sectional study	377 elderly patients with various comorbidities (aged from 66–97; 68.2% females; 89.2% European) EPIDOSO study.	Brazil	Apo A1 rs11216158 (XmnI), Apo A5 rs3135506 (S19W), ApoA5 rs662799 (−1131T>C)	Significantly higher TC in haplotype C/G (wild-type allele for XmnI and the minor allele for S19W) than haplotype C/C (wild type allele for XmnI and S19W). Significantly lower LDL in C-allele carrier than T-allele carrier in −1131T>C polymorphism.NS: between genetic polymorphism (XmnI and S19W) and TC, TG, VLDL, LDL and HDL.	[49]

ACS: acute coronary syndrome; EPIDOSO: Brazilian Elderly Longitudinal Study; GRS: genetic risk score; HC: healthy controls; HDL: high-density lipoprotein; HIV: human-deficiency virus; HW: Hardy–Weinberg; LDL: low-density lipoprotein; SNP: single-nucleotide polymorphism; TC: total cholesterol; TG: triglyceride.

### 2.2. Lipoprotein Lipase (LPL)

LPL, which is the major plasma triglyceride lipase, is attached to vascular endothelium via glycophosphatidylinositol (GPI)-anchored high-density lipoprotein-binding protein 1 (GPIHBP1) [50]. In lipid metabolism, LPL performs an important role in the hydrolysis of circulating triglyceride-rich lipoproteins, such as chylomicrons and VLDL [51]. Angiopoietin-like proteins (ANGPTL), which are a family of proteins with similar structure to angiopoietin, are also involved in lipoprotein metabolism, specifically related to LPL. These proteins are associated with the ability to inhibit LPL enzymatic activities and increases LPL cleavage. The inhibition of lipoprotein lipase activity by ANGPTL may lead to an increase in circulating lipid levels, especially triglyceride [52]. A structural interaction between ANGPTL8 and ANGPTL3 will form a complex that is a potent endogenous inhibitor of LPL [53,54]. In addition, another type of ANGPTL, namely ANGPTL4, has also been indicated to serve as a potent inhibitor of the LPL enzyme. ANGPTL4 can both attach and inactivate LPL complexed to GPIHBP1. The inactivation of LPL by ANGPTL4 causes reduction affinity of LPL towards GPIHBP1, causing dissociation [55].

Previous studies demonstrated that abnormal lipoprotein lipase, including deficiency and mutation, was firmly associated with the incidence of dyslipidemia, leading to consequences such as atherosclerosis and stroke [51]. The relationship between LPL and dyslipidemia in different populations are tabulated in Table 2. In a Saudi population, there were associations of LPL polymorphisms, namely HindIII with CAD, while LPL polymorphisms of PvuII and Ser447Ter demonstrated no association with CAD. Meanwhile, there were no significant values between the genotypes of the HindIII, PvuII and Ser447Ter polymorphisms in terms of TG, TC, HDL-c and LDL-c [56]. This result was also in parallel with a previous study conducted in a Macedonian population where the presence of LPL-PvuII polymorphism did not represent a statistically significant risk factor for CAD, thus, indicating a lack of association between this polymorphism and CAD [57]. LPL HindIII has also been investigated in an Iraqi smoking male population, which demonstrated associations between the lipid parameters of the smokers. Specifically, the genotypes of LPL HindIII polymorphism, H+H+ genotype group demonstrated significantly higher TG and VLDL-c concentrations while a significantly lower HDL-C concentration than those of the HeH- genotype [58]. Therefore, it seems that there were inconsistent findings in the LPL polymorphisms in terms of lipid parameters in the different populations.

In a more recent study, the association of other LPL gene polymorphisms, including rs1534649 and rs28645722 with plasma lipid levels, were examined. In this study, the T-allele of rs1534649 polymorphism demonstrated significantly low HDL-c, while the rs28645722 polymorphism revealed no association between plasma lipid levels [59]. Common polymorphism has affected the effectiveness of fibrate therapy, a commonly used drug for lowering TG and increasing HDL-c. The LPL synonymous rare variants were significantly associated with absolute HDL-c change and TG percent change in the patients treated with fibrate. This study indicated that individuals with dyslipidemia carrying rare synonymous variants within the LPL gene had an attenuated response to the fibrate therapy [60]. In summary, individuals carrying rs1534649 polymorphism may have higher risk in developing MetS, as it was associated with low HDL-C, which is one of the MetS components. Meanwhile, individuals who did not respond well to fibrate therapy might possess the rare synonymous LPL gene variants and alternative treatments should be considered.

## 3. The Role of Cholesterol-Related Genes in Obesity

### 3.1. Adiponectin

Adiponectin is a protein hormone secreted by adipose tissues and has a variety of metabolic effects on obesity, insulin sensitivity and atherosclerosis. Circulating adiponectin is independently correlated with increased cholesterol efflux, an initial step in the reverse cholesterol transport pathway whereby excess cholesterol in peripheral cells is packaged into high-density lipoprotein (HDL) [61]. Adiponectin upregulates the biosynthesis of the major apolipoprotein in HDL, namely as ApoA1 via the adiponectin receptor pathway. The upregulation of ApoA1 biosynthesis may increase HDL assembly in the liver, which demonstrates the important role of adiponectin in HDL metabolism and subsequently promotes protection against atherosclerosis [62,63]. Evidence indicates that low levels of adiponectin could be a useful marker for atherosclerosis, a condition of the buildup of fats, cholesterol and other substances in and on artery walls. Meanwhile, obesity has been recognized as a risk factor for atherosclerosis, in which serum adiponectin levels are also decreased in obese patients [61,64]. The serum levels are highly heritable and associated with the adiponectin gene (ADIPOQ) [65]. Single nucleotide polymorphisms (SNPs) such as rs1501299 (276G>T), rs266729 (_11377C>G) and rs822396 are among the most common polymorphisms of ADIPOQ and have been evaluated for their association with obesity (Table 3).

A positive correlation has been reported between the rs1501299 polymorphisms with obesity risk in North Indian Punjabi and Egyptian populations, which involved subjects with the mean age of 37.85 and 40.5 years old, respectively [66,67]. However, a previous study by Ogundele et al. demonstrated that the similar rs1501299 polymorphisms were not associated with obesity in a Nigerian population with the mean age of 22.2 years [68]. This discrepancy could be attributed to the age of the subjects involved in the studies, as aging is one of the factors that is associated with an increase in abdominal obesity [69]. Apart from that, these conflicting results could also be due to the differences in the genetic or epigenetics of the study populations. However, in the same study conducted in the Nigerian population, another ADIPOQ SNP, rs266729, was associated with increased measures of obesity involving BMI [68]. This may indicate that, within a population, there could be a contrary relation in ADIPOQ polymorphisms. This fact is also supported by Apalasamy et al., who reported that the ADIPOQ rs17366568 polymorphism showed significant association between obesity and genotype frequencies of the respective ADIPOQ polymorphism among a Malay population in Malaysia. However, in the same study, it was shown that another ADIPOQ rs3774261 polymorphism lacked association between the respective polymorphism and genotype frequencies [70].

Meanwhile, for another common ADIPOQ SNP, the rs822396 polymorphism, several studies have shown significant association with obesity in subjects with age more than 38 years old displaying metabolic features including increased BMI and hypoadiponectinemia [66,71,72]. Contradictorily, the ADIPOQ polymorphism showed no association with obesity-related variables, such as BMI and serum adiponectin levels, in young Jordanian women, consisting of normal weight, overweight and obese patients [73]. A study by Romero et al., which involved Mexican children, also demonstrated no associations with ADIPOQ polymorphisms in the overweight or obesity subjects [74]. These studies could indicate that age is a crucial factor in ADIPOQ polymorphism. Moreover, the ADIPOQ polymorphisms such as rs1501299, rs822396 and rs17366568 have shown positive association with obesity, with the sample population aged above 38 years old. However, most of the ADIPOQ polymorphisms were not associated with obesity when the sample population age was below 30 years old. These studies may indicate that the individuals carrying these ADIPOQ polymorphisms have a higher risk in getting obesity at an older age.

**Table 3:** The relationship between adiponectin and obesity.

Study Type	Model/Population	Country	Genes Involved	Findings	Reference
Cross-sectional study	Non-obese and obese Punjabi adults (*n* = 550; aged 37.85 ± 9.767 years).	North India	rs1501299rs822396	rs1501299 and rs822396 were positively associated with obesity.	[66]
Cross-sectional study	Non-obese and obese Egyptian adults (*n* = 197; aged 40.5 ± 19.5 years).	Egypt	rs1501299	rs1501299 was positively correlated with obesity risk.	[67]
Cross-sectional study	Underweight, normal, overweight and obese subjects (*n* = 107; aged from 18–30 years).	Nigeria	rs1501299	rs1501299 was not associated with obesity.	[68]
Cross-sectional study	Non-obese (*n* = 424; aged 46.17 ± 5.32 years) and obese (*n* = 150; aged 45.61 ± 7.37 years) subjects.	Malaysia	rs17366568 rs3774261	Rs17366568 polymorphism was associated with obesity and genotype frequencies.	[70]
Cross-sectional study	Men (*n* = 126; aged 47.0 ± 15.1 years) and women (*n* = 308, aged 47.2 ± 14.3 years).	Mexico	rs822396	rs822396 was associated with obesity.	[71]
Cross-sectional study	Subject with normal glucose tolerance or type 2 diabetes (*n* = 2200; aged 43 ± 14 years).	South India	rs822396	rs822396 was associated with obesity.	[72]
Cross-sectional study	Young women (*n* = 400; aged from 21–31 years old).	Jordan (North)	ADIPOQ gene SNPs (I146T and G276T)	ADIPOQ gene SNPs (I146T and G276T) showed no association with BMI.	[73]
Cross-sectional study	Normal and overweight/obese children (*n* = 1469; aged from 6–12 years old).	Mexico	rs182052, rs266729, rs2241766 and rs822393	No associations in ADIPOQ variants with the presence of overweight/obesity.	[74]

### 3.2. Peroxisome Proliferator-Activated Receptor-Gamma (PPARγ)

PPARγ is expressed in white and brown adipose tissue, the large intestine and spleen. However, its expression is the highest in adipose tissue and performs an essential role in the regulation of adipogenesis, energy balance, lipid biosynthesis and the transcriptional regulation of target genes involved in metabolic processes [75,76]. PPARγ is a master regulator of adipogenesis, a potent modulator of whole-body lipid metabolism and is also related with insulin sensitivity [75]. An energy imbalance that results in obesity can be caused by genetic or acquired changes in eating behavior, physical activity, energy storage and metabolism. Thus, genes implicated in energy metabolism and storage are suitable candidates to determine the susceptibility to obesity [77]. Two common isoforms of PPARγ, known as γ1 and γ2, are generated by alternative promoters and differential splicing of transcripts from the PPARγ gene on chromosome 3p25 [77]. PPARγ2 is the most crucial isoform in adipose tissue and is exclusively expressed [78].

For the PPARγ gene, a C>T polymorphism, which is located in the exon B and encodes the amino terminal polypeptide defining the PPARγ2 isoform, may contribute to a Pro12Ala substitution. The Ala allele has demonstrated a reduction in transactivating responsive promoter efficiency and the ability to stimulate adipogenesis in response to thiazolidinedione activation [79,80]. The findings of studies on the association of obesity with the PPARγ polymorphism have been inconsistent and are tabulated in Table 4. In a study by Darwish et al., which examines the correlation of PPARγ with obesity, it was shown that the mRNA expression of PPARγ was the most abundant in the serum of obese patients and statistically demonstrated strong positive correlation with the obesity variables, including the BMI, waist circumference and waist–hip ratio of obese patients [81].

The Pro12Aa gene substitution is also known as SNP rs1801282. In a study by Castro et al., performed in an overweight and obese Brazilian population, the SNP rs1801282 G-allele was associated with obesity in women, while the same allele was protective in men. Interestingly, these results suggested that the SNP rs1801282 is associated with obesity in a sex-specific manner, with the risk factor being in women [82]. Apart from that, the PPARγ gene variants have also been investigated in obese patients with ACS in a Turkish population. In this study, performed by Arat et al., they investigated the association of the PPARγ gene variant known as PPARγ proline with alanine substitution (Pro12Ala) in obesity [83], which is the most extensively examined in epidemiologic studies [84]. From the results, the PPARγ Pro12Ala polymorphism was significantly higher in obese patients with ACS, while the non-polymorphic genotype (proline/proline) was significantly higher in the control group. The results indicated that the PPARγ Pro12Ala polymorphism had the potential risk for ACS in obese individuals [83]. A previous study by Mehrad-Majd et al. investigated two common variants of PPARγ polymorphism, namely, the Pro12Ala and C1431T polymorphism with susceptibility to obesity in Iranian population. It was demonstrated that Pro12Ala had a correlation with predisposition to obesity-related markers, such as a higher body weight, waist circumference and waist–hip ratio. However, this effect did not show for the C1431T polymorphism of PPARγ2 gene [85].

In a study by Dujic et al., which involved three gene variants of PPARγ (Pro12Ala, C1431T, C681G) in a Bosnian population, all the gene variants were not significantly different between obese and normal subjects. Additionally, the Pro12Ala and C1431T variants had significantly lower BMI in the control subjects [86]. These results have contributed to an inconsistent finding between the gene variants of PPARγ, especially the Pro12Ala. However, the sample size used in this study was only 86, which could be considered a small sample size. According to a review by Simundic (2010), the sample size is dependent on the desired power of the study, level of significance and effect size. For instance, approximately 4000 patients and the same number of controls are required to detect the small effect size with 80% power [87].

Most of the previous studies discussed so far involved obesity in adults; however, Rahman et al. performed a study to determine the association between the Pro12Ala variant with BMI status among Malay children. The results showed a significant association between the Pro12Ala variant in the PPARγ gene with BMI status, suggesting the role of the Pro12Ala variant in the PPARγ gene in overweight Malay children [88]. This was supported by a cross-sectional study conducted among Malay adults demonstrating that the Pro12Ala of the PPARγ gene was closely associated with obesity, as shown by the Ala12 carriers had a significantly higher BMI [89]. Contradictory to the study by Rahman et al. in a population of children, a previous study by Dedoussis et al. among Greek pre-adolescents demonstrated no significant association between Pro12Ala PPARγ gene mutation and BMI status [90].

In a previous study by Ali et al., the Pro12Ala polymorphism in the PPARγ gene was investigated for its gender-specific effect on obesity risk in a Tunisian population. It was demonstrated that the Ala alelle polymorphism in the obese male patients, but not female, was positively correlated with obesity-related parameters, such as BMI and plasma leptin levels. Therefore, this study indicated that the Pro12Ala polymorphism of the PPARγ-2 gene was associated with obesity in men from Tunisian origin [91]. This result was supported by a previous study performed by Mattevi et al. in a Brazilian population whereby they demonstrated an association between the PPARγ Pro12Ala variant and BMI, with obesity male carriers of the Ala variant showing a higher BMI compared to the wild-type homozygotes. However, the effect of this polymorphism was not detected in women. Therefore, this study may also suggest that the Pro12Ala variant of the PPARγ gene probably had a gender-specific effect and was related to susceptibility to obesity in this population [77]. The Pro12Ala polymorphism of the PPARγ-2 gene is the most studied PPARγ gene regarding obesity, in which most studies discussed above have shown the positive correlation between this polymorphism and obesity risk. The significant correlation suggests that the Pro12Ala polymorphism had a regulatory role in obesity and, subsequently, in MetS development.

**Table 4:** The relationship between PPARγ and obesity.

Study Type	Model/Population	Country	Genes Involved	Findings	Reference
Case-control study	Normal, obese, diabetic non-obese and diabetic obese subjects (*n* = 100; from 57–59 years).	Egypt	PPARγ	In obese patients, PPARγ was positively correlated with BMI, waist circumference and waist–hip ratio.	[81]
Case-control study	Normal (*n* = 2240; 38.3 ± 10.4) and obese (*n* = 1844; 44.7 ± 12.3) Brazilian subjects.	Brazil	PPARγ SNP rs1801282-GG	PPARγ was strongly associated with overweight and obesity in women.	[82]
Case-control study	Obese (*n* = 50; 61.56 ± 8.92 years) and normal (*n* = 42; 60.34 ± 12.55 years) Turkish subjects.	Turkey	PPAR-γ Pro12Ala	The Pro12Ala polymorphism was positively correlated with common obesity variables, such as BMI, LDL-C and TG.	[83]
Case-control study	Normal (*n* = 150; 47 ± 7.76) and obese (*n* = 83; 48 ± 7.55) Iranian subjects.	Iranian	Pro12AlaC1431T	PPAR-*γ*2 gene polymorphism of Pro12Ala, but not C1431T, was correlated with predisposition to obesity-related markers.	[85]
Case-control study	Normal (*n* = 43; 41–51) and obese (*n* = 43; from 40–56) Bosnian subjects	Bosnia and Herzegovina	Pro12Ala, C1431T, and C681G	All three polymorphisms had no correlation with obesity-related markers.	[86]
Cross-sectional study	Malay children (*n* = 119; aged from 9–11 years)	Malaysia	Pro12Ala	Pro12Ala variant had a significant association with BMI among Malay children.	[88]
Cross-sectional study	Normal (*n* = 123; 33.59 ± 10.54) and obese (*n* = 94; 39.18 ± 9.97) subjects.	Malaysia	Pro12Ala	Pro12Ala variant had a significant association with BMI among Malay adults.	[89]
Cross-sectional study	Greek pre-adolescents children (*n* = 794; aged from 10–12 years).	Greece	Pro12Ala	Pro12Ala had no significant association with BMI.	[90]
Case-control study	Normal (*n* = 288; 48.77 ± 9.50 years) and obese (*n* = 387; 43.54 ± 12.27 years) subjects.	Tunisia	Pro12Ala	Pro12Ala was positively correlated with obesity-related parameters in the obese male patients.	[91]
Cross-sectional study	Obese men (*n* = 153; 41.3 ± 1.1) and women (*n* = 182; 38.7 ± 1.2).	Brazil	Pro12Ala	Pro12Ala variant was associated with higher BMI in male obese subjects than wild-type homozygotes. No such effect observed in female obese subjects.	[77]

BMI: body mass index; PPAR γ: peroxisome proliferator-activated receptor gamma; LDL-C: low-density lipoprotein; TG: triglyceride.

### 3.3. Leptin and Leptin Receptors

Obesity occurs when the body mass index (BMI) is greater than 30 kg/m^2^ [92]. It is characterized by a high proportion of body fat mass as well as an increase in leptin levels secondary to white adipose tissue accumulation [93]. Leptin is a peptide hormone encoded by the Lep^ob^ gene and is typically proportional to fat mass [94]. The role of leptin includes the regulation of food intake, body mass and fertility, and plays an important role in glucose homeostasis, immunity and angiogenesis [93,95]. Previous studies have shown that the circulating leptin level declines during fasting or energy restriction [96,97] but rises during refeeding, overfeeding and surgical stress [98,99]. These effects demonstrate the relation of the leptin signaling system and body mass maintenance. Moreover, leptin is also associated with lipid metabolism. For instance, systemic leptin increases lipolysis, a process of breaking down fats and other lipids in white adipocytes that also involve hormone-sensitive lipase (HSL) and nitric oxide (NO) synthase [100]. Leptin is also associated with the increased breakdown of triglycerides in skeletal muscle, oxidation of fatty acids in skeletal muscle and liver, and ketogenesis in the liver. Subsequently, this may result in a decreased size of adipose tissue accumulation and reduction in lipid content at the skeletal muscle and liver [100,101]. Leptin acts via its receptors, the leptin receptors that exhibit structural similarity to the class I family of cytokine receptors, including receptors for interleukins, colony-stimulating factor 3, growth hormone, prolactin and erythropoietin [93]. When fat cells increase, the leptin level increases proportionally and binds to leptin receptors in the hypothalamus, which subsequently sends signals to inhibit food intake and increase energy expenditure [98]. Several polymorphisms of both genes for leptin and leptin receptors have been studied in different populations for their potential association with obesity (Table 5). Among these variants, the LEP G2548A and Q223R single nucleotide polymorphisms (SNPs) have been studied in detail. These findings were replicated across different populations, revealing contradictory findings.

A study performed by Sahin et al. in a Turkish population, which involved a SNP in leptin consisting of a G to A substitution at nucleotide −2548 upstream (LEP G2548A), has been associated with increased leptin secretion in adipocytes, resulting in increased BMI [102]. In this study, the authors concluded that the substitution of LEP −2548 AA to AG genotypes are important predictors for increasing leptin levels and BMI in obese patients and could be a useful marker for obesity risk [102]. In a previous study performed by Boumaiza et al. in a Tunisian population, they also demonstrated the positive correlation for the LEP −2548 AA, whereby this gene variant could significantly increase the obesity risk. Subjects with 2548AA had significantly higher BMI, daily energy intake, waist circumference and abnormal lipid levels [103], which was closely related to the pathophysiology of obesity [104]. In the same study, the gene variant of the leptin receptor, LEPR Q223, had demonstrated positive correlation with obesity risk, similar to the leptin gene (LEP −2548 AA) [103]. Contradictorily, a study performed by Constantin et al. in a Romanian population showed that the polymorphism LEP G2548A and its receptor LEPR Q223R demonstrated no association with obesity. Additionally, they also found no association between the LEP 2548G/A polymorphism with common obesity-related variables, including BMI, fat mass and waist circumference [105]. The study by Pyrzak et al. showed a similar outcome, whereby no association between the LEPR Q223R polymorphism with leptin was identified among obese children in Poland [106].

Furthermore, the polymorphisms of the leptin and leptin receptors gene were also studied in patients taking antipsychotic agents [107]. In this study, mixed results were demonstrated between leptin and leptin receptors polymorphisms, whereby the LEP G2548A had no significant association with obesity. Meanwhile, for its receptors, LEPR Q223R and LEPR 223RR gene variants, gender influence was detected. The female patients demonstrated that LEPR 223QR and LEPR 223RR were associated with lower obesity risk, with genotype RR showing less average body weight. However, in males, no significant association was reported. Therefore, it seems that there is a gender influence for the LEPR Q223R polymorphism [107]. In a study by Duarte et al. in a Brazilian population, both polymorphisms of leptin (LEP G2548A) and its receptor (LEPR Q223R) were investigated for their relationship with obesity. In this study, there was a positive correlation association in LEP G2548A and LEPR Q223R variants in obese subjects, indicating that these polymorphisms can increase obesity risk [108].

Obese individuals were associated with a greater frequency of cardiovascular risk factors and higher morbidity and mortality rates [109]. Therefore, Arat et al. performed a study to observe the effect of the leptin receptor Gln223Arg polymorphism in obese patients with acute coronary syndrome (ACS). In this study, it was shown that the leptin Gln223Arg polymorphism did not demonstrate an association in obese patients with ACS in the Turkish population [83].

Therefore, leptin and its receptor have shown mixed results, with several studies demonstrating positive correlation and some showing a lack of correlation between the genes and obesity. The lack of association could be attributed to the complexity of obesity pathogenesis, which includes several genetic and environmental factors. As a recommendation, large studies that include the testing of multiple genes and polymorphisms in both obese and normal-weight subjects, which will provide epidemiologic data on dietary habits in different ethnic groups, are required to better understand the role of leptin in regulating weight in human populations.

**Table 5:** The relationship between leptin, leptin receptor and obesity.

Study Type	Model/Population	Country	Genes Involved	Findings	Reference
Cross-sectional study	Obese (*n* = 47; 44.28 ± 9.40 years) and healthy volunteers (*n* = 48; 41.96 ± 10.05 years).	Turkey	LEP c.−2548 G>A	LEP c.−2548 G>A polymorphism was positively correlated with serum LEP and BMI.	[102]
Cross-sectional study	Non-obese (BMI < 30 kg/m^2^) (*n* = 169; 47.86 ± 11.17 years) and obese (*n* = 160; 42.16 ± 14.26 years) volunteers.	Tunisia	LEP G2548A LEPR Q223R	Both LEP 2548AA and LEPR Q223R were positively correlated with obesity risk.	[103]
Cross-sectional study	Obese (*n* = 108; 51 ± 7 years) and non-obese (*n* = 94; 42 ± 11 years) Romanian subjects.	Romania	LEP G2548A andLEPR Q223R	No association between the LEP G2548A and LEPR Q223R polymorphisms and obesity	[105]
Case-control study	Obese (*n* = 101) and normal (*n* = 141) children (from 12–18 years)	Poland	LEPR Q223R	LEPR Q223R polymorphism was not associated with obesity.	[106]
Cross-sectional study	Psychotic disorder patients (*n* = 200; from 18–65 years) on an atypical antipsychotic for at least 3 months.	Netherlands	LEP G2548A, LEPR Q223R and LEPR 223RR	LEPR Q223R and LEPR 223RR genotypes were negatively correlated with obesity in women, but not in men. No significant association was found between the LEP 2548G/A polymorphism and obesity.	[107]
Cross-sectional study	Obese (*n* = 200; 32.0 ± 9.7 years) and non-obese (*n* = 150; (42.3 ± 12.1 years) Brazilian subjects.	Brazil	LEP G2548A LEPR Q223R	Both the LEP G2548A and LEPR Q223R variants were related to the increase in obesity risk.	[108]
Case-control study	Obese (*n* = 50; 61.56 ± 8.92 years) and normal (*n* = 42; 60.34 ± 12.55 years) Turkish subjects.	Turkey	LEPR Gln223Arg	LEPR Gln223Arg was not associated with obesity in ACS patients.	[83]

ACS: acute coronary syndrome; BMI: body mass index; LEP: leptin; LEPR: leptin receptor.

## 4. The Role of Cholesterol-Related Genes in Hyperglycemia

### 4.1. Adiponectin

Adiponectin is a cell-signaling peptide produced by adipocytes and is involved in regulating glycaemia status and insulin responsivity. It is encoded by the Adipose Most Abundant Gene Transcript 1 (APM1) gene. An abnormal adiponectin secretion could be affected by single-nucleotide polymorphisms (SNPs) of the APM1 gene, in which the most commonly studied SNPs in the APM1 gene are rs2241766 (T45G) and rs1501299 (G276T) [110]. Table 6 summarizes the relationship of adiponectin and its polymorphism with hyperglycemia. Human plasma levels of adiponectin have frequently been observed to have a strong inverse correlation with an increase in visceral adiposity [111] and insulin resistance [112,113]. These initial observations, thus, suggest that adiponectin has an important role in insulin actions and low levels of adiponectin in blood, known as hypoadiponectinemia, may result in insulin resistance and diabetes mellitus. A generation of adiponectin-deficient mice equally proved the association with heterozygous genotype (adipo(+/−) mice showing a mild insulin resistance phenotype, while homozygous genotype (adipo(−/−)) mice were observed to have more severe insulin resistance and glucose intolerance [114]. Further supporting these observations, genetic polymorphism in the human adiponectin gene had been proposed to be part of the genetic contributor of metabolic syndrome, including an impaired glucose tolerance state and diabetes mellitus [115].

The physiological effect of Adiponectin is mostly mediated via two specific receptors, AdipoR1 and AdipoR2, which are involved in the activation of the AMPK and PPARα pathways, respectively. Fascinatingly, these two receptors acted in different ways in their responses to Adiponectin [116]. Knock-out of the AdipoR1 receptor in mice was observed to increase insulin sensitivity and decrease their glucose tolerance, while an AdipoR2 knock-out mouse model showed better sensitivity to glucose and insulin, thus, maintaining a normal body weight despite a high fat diet [116].

The administration of Troglitazone, a ligand to both PPARα and PPARγ, increases plasma adiponectin levels among patients with impaired glucose tolerance when compared to those receiving a placebo [117]. In an in vivo animal study, upon treatment of obese diabetic mice with thiazolidinedione (TZD), an antidiabetic agent that improves systemic insulin sensitivity, adiponectin levels were shown to be up-regulated both at the transcript level in the adipocyte and circulating plasma concentration [118]. TZD is a PPARγ agonist that activates AMPK in muscle and liver and has been shown to impede gluconeogenic pathways via down-regulation of G6Pase and phosphoenol-pyruvate carboxykinase. Another study using adiponectin null mouse, TZD effects on glucose homeostasis is dysregulated due to the failure of AMPK pathway activation, which led to impaired glucose tolerance [119].

As adiponectin levels are shown to be closely associated with glucose homeostasis, the clinical application of plasma adiponectin levels as an independent predictor of Type 2 diabetes mellitus has been studied, revealing positive predictive associations [120,121]. In summary, decreased plasma adiponectin levels and carrying SNPs at certain positions (−4034, −3964 and 276) are associated with diabetic and impaired glucose tolerance as well as an increased insulin resistance index, which could increase the risk of metabolic syndrome.

**Table 6:** The relationship between adiponectin gene and its polymorphism with hyperglycemia.

Study Type	Model/Population	Country	Genes Involved	Findings	Reference
Human	Plasma adiponectin were measured in 87 nonobese subjects and 57 obese subjects.	Japan	APM1	A strongly significant inverse correlation between plasma adiponectin and BMI in both genders.	[111]
Human	Plasma Adiponectin levels in 183 diabetic and 82 healthy controls.	Japan	APM1	Plasma adiponectin was statistically lower in diabetic compared to nondiabetic subjects in both gender.	[112]
Human	Plasma Adiponectin levels in 17 diabetic, 25 impaired glucose tolerance, 79 normal glucose tolerance test.	USA (Caucasian and Pima Indians)	APM1	Plasma adiponectin was significantly lower in diabetic and impaired glucose tolerant subjects in comparison to normal glucose tolerant subjects.	[113]
Animal	Generation of adiponectin-deficient mice (heterozygous genotype (adipo(+/−) and homozygous genotype ((adipo(−/−)), looking at the development of glucose intolerance and/or diabetic state.	Japan	AdipoQ	adipo (−/−) mice showed significant insulin resistance with presence of glucose intolerance, while the heterozygous phenotype (adipo (+/−)) mice showed a milder insulin resistance.	[114]
Human	Genotyping for polymorphisms in adiponectin gene in 480 nondiabetic and 384 diabetic participants.	Japan	APM1 SNPs	SNPs at positions 45 and 276 were identified to be statistically significant differences between the groups. SNPs at positions −4034, −3964 and 276 were associated with an increased in insulin resistance index.	[115]
Human	Human: 29 subjects with impaired glucose tolerance randomized to receive Troglitazone or placebo. Plasma adiponectin measured at baseline and after 12 weeks of intervention.	Japan	APM1	Administration of Troglitazone significantly increased plasma adiponectin level among human subjects with impaired glucose tolerance state, while those receiving placebo showed no change in their plasma adiponectin level.	[117]
Mice	Plasma adiponectin level after treatment of obese-diabetic mice with TZD.		AdipoQ	Increment of Adiponectin level (transcript and plasma level) upon TZD treatment.	[118]
Mice	Effect of TZD-mediated glucose tolerance in Adiponectin-null mice.		AdipoQ	Effect of TZD-mediated glucose tolerance was attenuated in adiponectin-null mice, highlighting adiponectin action through activation of the AMPK pathway via PPARγ ligand.	[119]
Human	Plasma adiponectin level in 91 subjects with impaired glucose tolerance, with reassessment after 1 year revealed 25/91 subjects subsequently developed diabetes.	India	APM1	Mean baseline adiponectin level was higher in non-diabetic in comparison to diabetic patients.	[120]
Human	Baseline plasma adiponectin measurement in 27,548 subjects followed up from 2–3 years, which identified 192 with medically confirmed T2DM.	USA	APM1	High plasma adiponectin concentration is independently and strongly link with attenuated risk of T2DM in healthy subjects.	[121]

APM1: Adipose Most Abundant Transcript 1 Gene; AMPK: AMP-activated protein kinase; BMI: body mass index; PPAR γ: peroxisome proliferator-activated receptor gamma; SNP: single nucleotide polymorphism; T2DM: Type 2 diabetes mellitus; TZD: Thiazolidinedione.

### 4.2. Apolipoprotein

Serum lipoproteins may affect tissue insulin sensitivity both directly and indirectly via the activity of major lipoproteins. For instance, apolipoproteins CIII (ApoC3) and E (ApoE), which can modulate whole-body insulin sensitivity by regulating the plasma triglycerides and nonesterified fatty acids (FFAs), transfer to tissues [122]. Therefore, apolipoprotein plays a key role in insulin sensitivity that is closely related with hyperglycemia.

Single nucleotide polymorphisms of the apolipoprotein gene have contributed to mixed results regarding the association of the genetic effects on type 2 diabetes mellitus risk (Table 7). In a previous study by Seo et al. (2021), conducted in a Korean population, the APOE polymorphisms including rs7412 and rs429358 had no significant association with T2DM [123]. However, a previous study by Alharbi et al. (2014) demonstrated that the similar ApoE polymorphism (rs7412 and rs429358) was significantly associated with T2DM in a Saudi population with the ɛ2 and ɛ4 alleles [124]. The ApoE polymorphism has also been examined for its association with anti-hyperglycemic therapy, whereby significant improvement in cardiometabolic outcomes among APOE4 carriers taking a metformin and metformin-sulfonylurea combination was observed. However, no significant improvement was observed in APOE4 carriers taking other hypoglycemic agents, such as insulin, thiazolidinediones and dipeptidyl peptidase-4 inhibitors. This case-control study, which was conducted in a South Asian population, particularly in a Punjabi-Sikh population, indicated that the APOE polymorphism played a role in the therapeutic effects of anti-hyperglycemic agents [125].

In summary, the APOE polymorphism also had inconsistent results in the relationship with hyperglycemia in different populations. Additionally, as stated earlier, the APOE polymorphism carriers taking metformin, the first-line therapy for T2DM, may have protection against cardiometabolic outcomes which is the ultimate risk in metabolic syndrome.

## 5. The Role of Cholesterol-Related Genes in Hypertension

### 5.1. Lipoprotein Lipase (LPL)

Lipoprotein lipase (LPL) is a water-soluble enzyme involved in the hydrolysis of circulating triglycerides carried by chylomicrons and very low-density lipoproteins (VLDL), releasing free fatty acids for energy metabolism and storage in adipose tissues. The LPL gene is located on chromosome 8p22 and over 100 mutations have been identified on this gene [126]. Most of the mutations in the LPL gene result in the loss of enzymatic function, except for Ser447Ter (a variant with non-synonymous mutation in the exon 9), which is associated with lipolytic function and anti-atherogenic properties [127]. LPL Ser447Ter is the most common polymorphism of LPL and has been assessed for its association with blood pressure and risk of hypertension. Previous studies have demonstrated conflicting findings, suggesting both positive and negative associations between LPL polymorphism and hypertension (Table 8). In apparently healthy populations, individuals carrying the LPL Ser447Ter variant had lower systolic, diastolic and pulse pressure [128,129,130]. On the contrary, a positive relationship was observed between LPL Ser447Ter with hypertension in subjects displaying MetS features [131,132,133,134,135]. Researchers postulated that the discrepancy might be attributed to small sample size, diverse genetic background and the different inclusion/exclusion criteria set for each study. However, it seems that the Ser447Ter variant is the most studied LPL gene regarding hypertension, in which the inconsistent results were reported in different populations.

### 5.2. Cholesteryl Ester Transfer Protein (CETP)

Cholesteryl ester transfer protein (CETP) is a 74 kDa glycoprotein produced by human liver and adipose tissues, secreted into the circulation, and associated with HDL particles. It allows redistribution of cholesteryl esters and triglycerides between VLDL, LDL and HDL. Low levels of CETP promotes HDL formation, thus, is associated with a lower risk of cardiovascular diseases. The association between CETP and hypertension has been explored in animals and humans (Table 9). The CETP-overexpressed spontaneous hypertensive rats fed with standard rat chow had markedly elevated triglycerides, systolic blood pressure, reduced HDL cholesterol and accelerated fatty liver development [137]. In humans, the relationship appeared to be heterogenous. The circulating plasma CETP activity was not related to the change in systolic and diastolic blood pressure in a large community-based study with 1307 participants free from hypertension and cardiovascular disease with a mean age of 48 years old [138]. A cohort study consisting of older adults aged 95 years old and above showed that a reduced CETP level was correlated with lower blood pressure. In this study, participants taking blood pressure medication were included in the analysis, thus, the high blood pressure may be masked by the use of anti-hypertensive medications [139].

The effects of CETP inhibitors on blood pressure have also been investigated. Torcetrapib is the first CETP inhibitor developed to treat hypercholesterolemia and prevent cardiovascular disease. Inhibition of CETP with torcetrapib increased plasma HDL cholesterol levels but is also associated with increased blood pressure. In in vivo models of small and large animals, treatment with torcetrapib caused an acute increase in blood pressure in mice [140], rats [141] and monkeys [142]. CP-532623, a CETP inhibitor with a close structural analogue of torcetrapib, exhibited similar effects in increasing blood pressure of cynomolgus monkeys and healthy human volunteers [142]. The mechanism underlying the blood pressure-rising effects of torcetrapib and CP-532623 were contributed to by increased aldosterone and cortisol synthesis in the adrenal gland and endothelin expression in the arterial wall, but independent of CETP inhibition [140,143]. Evacetrapib and anacetrapib are potent and selective CETP inhibitors with HDL cholesterol-rising action but without torcetrapib-like off-target effect [138]. In vitro evidence showed that evacetrapib failed to induce production of aldosterone and cortisol in human adrenal corticocarcinoma cells [141]. A double-blind, randomized, placebo-controlled study found that there was no correlation between exposure to evacetrapib and blood pressure in healthy volunteers. No clinical difference was observed in the plasma renin activity and cortisol level between those receiving evacetrapib or placebo [144]. Anacetrapib exerted a dose-dependent effects in increasing HDL-C and decreasing LDL-C in patients with dyslipidaemia but did not influence their blood pressure [145,146]. Similarly, no alteration in 24-h ambulatory blood pressure was observed in healthy individuals [145].

In brief, a low level of CETP or its inhibition are unlikely to correlate with increased blood pressure. The blood pressure-rising effects of CETP inhibitors vary among subtypes, whereby the effects were absent in the newer CETP inhibitors, thus, suggesting this phenomenon was independent of CETP inhibition.

**Table 9:** The relationship between CETP and blood pressure.

Study Type	Model/Population	Intervention (Dose, Route, Frequency, Duration)	Findings	Reference
Animal study	Human CETP-transgenic strain on spontaneous hypertensive rats.	-	Mice with transgenic overexpression of CETP had elevated systolic blood pressure, triglycerides and reduced HDL cholesterol.	[137]
Cohort study	Older adult (*n* = 521; aged ≥ 95 years).	-	Reduced CETP level was associated with lower blood pressure.	[139]
Community-based study	Participants free from cardiovascular disease (*n* = 1307; mean age: 48 years).	-	Plasma CETP activity was not associated to systolic and diastolic blood pressure.	[138]
Animal study	C57BL/6NTac mice.	Torcetrapib or anacetrapib (10 mg/kg, i.v., 30 min)	Torcetrapib increased MAP, aldosterone and corticosterone, but not in anacetrapib.	[140]
Animal study	Male and female cynomolgus monkey.	CP-532623 (420 mg/kg, single dose, oral); torcetrapib (10 and 100 mg/kg/day, oral, 2 weeks)	Treatment with CP-532,623 or torcetrapib elevated systolic and diastolic blood pressure as compared to treatment with vehicle.	[142]
Randomized, double-blind, placebo-controlled, single-dose study	Healthy male subjects (*n* = 56; aged from 18–55 years).	CP-532623 (100 and 300 mg, oral, single dose)	Treatment with CP-532,623 increased HDL cholesterol, systolic and diastolic blood pressure.	
Animal study	Male obese Zucker diabetic rats.	Torcetrapib (60 mg/kg, oral, single dose) or evacetrapib (160 mg/kg, oral, single dose)	Torcetrapib increased MAP, but not in evacetrapib.	[141]
In vitro	Human adrenal cortical carcinoma H295R cells.	Evacetrapib (0.001–10 μM)	Evacetrapib did not induce aldosterone or cortisol synthesis.	
Placebo-controlled study	Healthy volunteer (*n* = 76; aged 36.8 ± 9.6 years).	Evacetrapib (10–600 mg/day, oral, 15 days)	Evacetrapib did not affect 24-h ambulatory systolic and diastolic blood pressure.	[144]
Double-blind, randomized, placebo-controlled study	Patients with dyslipidemia (*n* = 50; aged from 18–75 years).	Anacetrapib (0–300 mg/kg, oral, 28 days)	Anacetrapib increased HDL cholesterol and decreased LDL cholesterol.	[145]
	Healthy participants (*n* = 22; aged from 45–75 years).	Anacetrapib (150 mg/kg, oral, 10 days)	Anacetrapib did not alter blood pressure.	
Multicenter, randomized, placebo-controlled, double-blind, dose-ranging study	Patients with dyslipidemia (*n* = 407; aged from 20–75 years).	Anacetrapib (10–300 mg/day, oral, 8 weeks)	Anacetrapib did not alter blood pressure and electrolytes.	[146]

CETP: Cholesteryl ester transfer protein; HDL: high-density lipoprotein; MAP: mean arterial pressure.

### 5.3. Apolipoprotein

Apolipoprotein is a plasma lipoprotein component that binds and transport blood lipids to various body tissues for metabolism and utilization. It can be divided into many subclasses including A, B, C, D, E, F, H, L, M, N and R. The mutation of apolipoprotein, particularly the different formations of allelic polymorphisms, results in different phenotypes of apolipoprotein, thus, affecting the metabolism and utilization of blood lipids [147]. The perturbation of lipid metabolism triggers the development of hypertension, metabolic and cardiovascular diseases. The relationship between various apolipoproteins and hypertension has been validated (Table 10).

Apolipoprotein A1 (apoA1) is the major component of HDL cholesterol and acts as an anti-atherogenic factor by promoting fat efflux through reversal cholesterol transport from tissue to liver for excretion. In a case-control study, there was no significant difference in the serum apoA1 level between the patients with essential hypertension and the matched controls. However, the correlation analysis revealed a negative association between the serum apoA1 level with blood pressure [148]. A large cohort indicated the level of apoA1 was lower in women with hypertension as compared to healthy women after eight years [149]. The polymorphisms in apoA1 are also closely associated with hypertension. The most common polymorphisms of apoA1 are −75 G/A and +83 C/T, representing the guanine (G) allele to alanine (A) allele substitution at −75 bp of the apoA1 gene and the cytosine (C) allele to thymine (T) allele substitution at +83 bp of the apoA1 gene, respectively. Both apoA1 polymorphisms increase transcription efficiency, which has been associated with a higher HDL cholesterol level. The −75 G and +83 C alleles were significantly associated with hypertension in the elderly [150]. Similar outcomes were detected in a case-control study, with a lower frequency of the CT genotype and T allele in subjects with hypertension among a Chinese population [151]. However, another study demonstrated that the presence of GA (at the −75 bp site) and CT (at the +83 bp site) heterozygosity in hypertensive patients, while the most predominant genotype observed in the control group were GG (at the −75 bp site) and CC (at the +83 bp site) homozygosity in an Indian population. Moreover, heterozygosity in the apoA1 gene was associated with higher odds of developing essential hypertension [152]. The authors postulated that the difference in genotypic frequencies obtained from the two case-control studies might be due to different sample sizes and genetic backgrounds in different populations.

Apolipoprotein B (apoB) is a main structural component of atherogenic lipoproteins (including chylomicrons, LDL, IDL and VLDL particles) that facilitates fat and cholesterol transportation to the peripheral tissues [153]. High levels of apoB correspond to high levels of LDL and VLDL cholesterol, which are often related to a higher risk of cardiovascular diseases. The association between apoB with the risk of hypertension was analyzed in middle-aged and older women. Women with hypertension had higher apoB levels [149,154]. In a recent cohort, normotensive Japanese–American men and women (*n* = 233) aged 46.4 ± 11.0 years were recruited to investigate whether plasma apoB concentration predicted the risk of hypertension over a 10-year study period. The baseline apoB concentration was positively correlated with the incidence of hypertension [155]. A case-control study indicated that there was a rise in serum apoB100 and apoB100:apoA1 ratio in the patients with essential hypertension, which was not seen in the matched control counterparts. Correlation analysis also found a positive association between apoB100 levels with systolic and diastolic blood pressure [148].

Apolipoprotein C3 (apoC3) is a low molecular weight protein and exchangeable surface component found on chylomicrons, VLDL, LDL and HDL. It acts as an inhibitor of LPL activity and is an important mediator in promoting atherogenicity [156,157]. However, limited information is available on the effects of apoC3 genetic variation in predicting the development of hypertension. The association between apoC3 with hypertension onset was tested in Korean adults aged from 40–69 years over a 9.8-year follow-up. The findings showed that the genotype effects of apoC3 on hypertension risk were evident [158].

Apolipoprotein E (apoE) exists as a part of chylomicron remnants VLDL, IDL and HDL, which has the ability to protect against atherosclerosis by promoting lipid clearance [159]. Deficiency in the apoE gene resulted in higher blood pressure, lipid accumulation and plasma lipid profile (total cholesterol, triglycerides, LDL and HDL) in mice fed an atherogenic diet [160]. There are three common alleles (ε2, ε3 and ε4) for apoE with six different genotypes (ε2/2, ε2/3, ε2/4 ε3/3, ε3/4 and ε4/4). Amongst these, ε3/3 is the wild-type, while others are the mutant types. Extensive studies have been performed to investigate the relationship between apoE polymorphism and hypertension. Meta-analysis performed on 28 case-control studies revealed that the ε2 allele and ε2/2 and ε2/3 genotypes can be protective factors; meanwhile, the ε4 allele and ε3/4 and ε4/4 genotypes can be risk factors for hypertension [161,162].

The circulating apolipoprotein L1 (apoL1) level is closely correlated with the plasma triglyceride level [163]. Apart from being a minor component of HDL cholesterol, apoL1 plays an essential role in triggering inflammatory response [164], autophagy [165] and innate immunity [166]. Overwhelming inflammation, excessive autophagy and the accumulation of immune cells in blood vessels can lead to the pathogenesis of hypertension and resulting heart diseases [167,168]. A study by Nadkarni et al. reported that apoL1 risk alleles were associated with the increase in systolic blood pressure and earlier diagnosis of hypertension in young African American adults [169]. Recent research with prospective cohort study design revealed no changes in systolic and diastolic blood pressure between apoL1 high risk and low risk groups over a period of 7.8 years of follow-up [170]. Inconsistent findings and limited evidence serve as the potential research gap, which require similar large cohorts to be conducted to draw a conclusion on the possible link between apoL1 and hypertension.

**Table 10:** The relationship between apolipoprotein and blood pressure.

Study Type	Model/Population	Findings	Reference
Case control study	Patients with essential hypertension and matched controls (*n* = 110; aged from 30–50 years).	No change in apoA1 but rises in apo B100 and apo B100/apoA1 ratio were observed in the hypertensive patients. Apo A1 was negatively correlated but apoB100 was positively correlated with systolic and diastolic blood pressure	[148]
Cohort study	Initially healthy women (*n* = 17,527; aged ≥ 45 years).	ApoA1 level was lower but apoB level was higher in women with hypertension than those without hypertension after 8 years.	[149]
Cohort study	Male and female subjects (*n* = 334; aged 79.54 ± 5.15 years).	APOA1 polymorphisms (−75 G and +83 C-alleles) were associated with hypertension.	[150]
Case control study	Hypertensive patients (*n* = 104; aged 47 ± 10 years) and healthy controls (*n* = 167; aged 36 ± 11 years).	Lower frequency of the CT genotype and T-allele of apoA1 gene in the hypertensive patients.	[151]
Case control study	Patients with essential hypertension and control subjects (*n* = 100, aged from 35–60 years).	GA (−75 bp) and CT (+83 bp) heterozygosity in the apoA1 gene was observed among the hypertensives.	[152]
Cross-sectional study	Postmenopausal women (*n* = 242; aged from 35–70 years).	Women with hypertension had higher apoB.	[154]
Cohort study	Normotensive Japanese Americans (*n* = 233; aged 46.4 ± 11.0 years).	The baseline apoB level was positively associated with the odds of incident hypertension over 10 years.	[155]
Community-based cohort study	Korean men and women (*n* = 5239; aged from 40–69 years).	APOC3 was associated with the risk of hypertension.	[158]
Animal experimentation	ApoE^−/−^ CYP1B1^−/−^ mice.	Blood pressure, lipid accumulation and plasma lipids were increased in the ApoE−/− mice fed with atherogenic diet. The inhibition of CYP1B1 minimized the changes in ApoE^−/−^ mice fed with atherogenic diet.	[160]
Case control study	Hypertensive patients (*n* = 94; aged 57.93 ± 12.95 years) and healthy controls (*n* = 102; aged 63.81 ± 11.19 years).	Apolipoprotein E3/4 genotype was higher in the hypertensive group.	[162]
Cohort study	African Americans (*n* = 5204; age not mentioned).	ApoL1 risk alleles was associated with higher systolic blood pressure and earlier hypertension diagnoses.	[169]
Prospectivecohort study	Men and women without baseline clinical cardiovascular disease (*n* = 1619; aged from 45–84 years).	No longitudinal influences in systolic, diastolic and pulse pressure by apoL1 risk status among the United States population.	[170]

ApoA1: apolipoprotein-A1; ApoE: apolipoprotein-E; ApoL1: Apolipoprotein-L1.

## 6. Perspectives

The pathophysiology of MetS is multifaceted, with sedentary lifestyle, overnutrition and genetic factors that clearly interact to produce the syndrome. In this context, the modification of environmental factors and the alternation of genetic factors can be a holistic approach for MetS management. The current evidence indicates that adiponectin is positively associated with obesity in elderly populations, but such association was not seen in younger populations. PPARγ was directly associated with obesity, meanwhile the lack of correlation found between the leptin-related gene and obesity was mainly due to distinct dietary habits across populations. Higher adiponectin levels were also shown to be directly associated with hyperglycemic condition. The polymorphism of LPL was associated with higher blood pressure in MetS subjects, but this outcome was not seen among healthy individuals. A circulating CETP level was unlikely to cause hypertension. However, the subtypes of CETP inhibitors exert different impacts on aldosterone and cortisol synthesis, thus, affecting the blood pressure differently. For apolipoproteins, apoA1 and apoE polymorphism were closely associated with hypertension. In addition, apoB was positively correlated with hypertension and dyslipidemia, whereas apoC1 was also positively correlated with hypertension. The listed cholesterol-related genes in this review may serve as the basis for future diagnosis, prognosis and therapy for MetS. We addressed the limitation of the current review. Firstly, our discussion was limited to the role of selected cholesterol-related genes in MetS conditions. Secondly, MetS occurs with the presence of multiple metabolic abnormalities, in which the role of cholesterol-related genes may differ from one component to another. Thirdly, this review provides an overview on the direct effects of cholesterol-related genes in each MetS component, with the mechanism of action not discussed any further. Although dyslipidemia is a major constituent of MetS, and the treatment of dyslipidemia could be a potential management, diabetes and hypertension in MetS should be also prevented.

## 7. Conclusions

MetS is a complex trait resulting from the interplay between environmental factors and underlying genetic susceptibility factors. However, the current evidence reveals a heterogenous relationship between cholesterol-related genes and MetS, which awaits further investigation using controlled experimental settings in animals and larger sample sizes in humans. The use of medications in recruited participants should also be a point of consideration in future studies for better understanding the association between cholesterol-related genes and MetS.

## Figures and Tables

**Table 2:** The relationship between specific polymorphisms of lipoprotein lipase and lipid parameters.

Study Type	Model/Population	Country	Genes Involved	Findings	Reference
Case-control	120 CAD patients (age 61.94 ± 10.78 years, 65 control (age 49.02 ± 19.73) subjects.	Saudi Arabia	LPL-*Hin*dIII, LPL-*Pvu*II and LPL-Ser447Ter	*Hin*dIII polymorphism was associated with CAD, while no association of *Pvu*II and Ser447Ter polymorphisms with CAD.	[56]
Case-control	109 CAD patients (mean age 59.4), 32 normal coronarographic findings (mean age 57.9).	Macedonia	LPL-PvuII polymorphism	LPL-PvuII polymorphism had no association with CAD.	[57]
Case-control	185 apparently healthyIraqi males (90 smokers age and 95 non-smokers age 42.76 ± 8.22 years).	Iraq	LPL-Hind III	LPL-Hind III had associations between thepolymorphism and the lipid parameters of smokers.	[58]
Observational study	486 adults (mean age: 29.87 ± 13.02).	Kuwait	LPL polymorphisms including rs1534649 and rs28645722	T-allele of rs1534649 had a significant association with dyslipidemia risk. No associations betweenrs28645722 and plasma lipid levels.	[59]
Randomized, double-blind, active-controlled study	2385 European–American with mixed dyslipidemia.	USA	Rare LPL gene variants	Synonymous variants of LPL gene were associated with an attenuated response to FA therapy.	[60]

CAD: coronary artery disease; FA: fibric acid; LPL: lipoprotein lipase.

**Table 7:** The relationship between apolipoprotein and hyperglycemia.

Study Type	Model/Population	Country	Genes Involved	Findings	Reference
Case-control study	1436 subjects with 352 T2DM patients (59.81 ± 7.78 years) and 1084 unaffected controls (54.93 ± 9.52 years).	Korea	*APOE* polymorphisms including rs7412 and rs429358	No significant associations among the *APOE* 6 tagging SNPs, ε genotypes and haplotypes with T2DM susceptibility.	[123]
Case-control study	898 subjects with 438 T2DMpatients (53.5 ± 10.78 years) 460 controls (46.13 ± 7.77).	Saudi Arabia	*APOE* polymorphisms including rs7412 and rs429358	APOE alleles (ε2 and ε4) had a significant association T2DM in terms of insulin and HomaIR parameters.	[124]
Case-control study	3564 subjects with 1956 diabetic patients (55.4 ± 11.2 years), 1608 controls (50.0 ± 14 years).	India	APOE polymorphisms	*APOE*4 carriers had significant association with regards to anti-hyperglycemic therapy including metformin and metformin-SU combination therapy.	[125]

T2DM: type 2 diabetes mellitus; HomaIR: Homeostatic Model Assessment of Insulin Resistance; SNP: single nucleotide polymorphism; SU: sulfonylureas.

**Table 8:** The relationship between lipoprotein lipase and blood pressure.

Study Type	Model/Population	Country	Genes Involved	Findings	Reference
Cross-sectional study	Healthy volunteers (*n* = 1583; aged from 29–55 years).	France	Ser447Ter	Ser447Ter variant was negatively correlated with systolic and pulse pressure.	[130]
Cross-sectional study	Female twin subjects (*n* = 893; aged 36.2 ± 8.8 years).	China	Ser447Ter	Ser447Ter variant was negatively correlated with systolic and diastolic blood pressure.	[129]
Cohort study	Ser447Ter (*n* = 91; aged 42 ± 14 years) and non-Ser447Ter carriers (*n* = 316; aged 41 ± 16 years).	Canada	Ser447Ter	Ser447Ter variant was negatively correlated with systolic and diastolic blood pressure.	[128]
Community-based study	Subjects with essential hypertension (*n* = 904; aged from 35–69 years).	China	Ser447Ter	Ser447Ter variant was positively associated with systolic and pulse pressure.	[134]
Case-control study	Young-onset hypertension patients (*n* = 60; aged 46.8 ± 7.3 years) and normal controls (*n* = 60; aged 47.7 ± 7.2 years).	Taiwan	Ser447Ter	Ser447Ter variants were positively associated with persistent hypertension.	[131]
Case-control study	Normotensive (*n* = 50; aged 46.2 ± 8.2 years) and hypertensive (*n* = 50; aged 49.1 ± 9.5 years) patients.	Dubai	Ser447Ter	Ser447Ter variants was positively associated with risk of developing hypertension.	[133]
Case-control study	Healthy and MetS subjects (*n* = 401, age not mentioned).	China	Ser447Ter, HindIII	HindIII and Ser447Ter variants were positively associated with diastolic and diastolic blood pressure.	[132]
Family-based association studies	Subjects with obesity (*n* = 90; aged ≥ 18 years).	Mexico	Ser447Ter, HindIII	HindIII variant was positively associated with diastolic blood pressure. Ser447Ter variant was positively associated with systolic and diastolic blood pressure.	[135]
Cohort study	Normotensive (*n* = 501; aged 53.7 ± 9.1 years) and essential hypertensive (*n* = 497; aged 53.5 ± 9.3 years) subjects.	China	Ser447Ter, IVS4-214C>T, 7754C>A	No association between single polymorphism and hypertension or blood pressure levels. More powerful haplotypes analysis suggested an association between LPL gene and hypertension.	[136]

LPL: lipoprotein lipase.

## Data Availability

Not applicable.
